# Mitochondrial metabolic reprogramming in diabetic kidney disease

**DOI:** 10.1038/s41419-024-06833-0

**Published:** 2024-06-24

**Authors:** Xiaoting Fan, Meilin Yang, Yating Lang, Shangwei Lu, Zhijuan Kong, Ying Gao, Ning Shen, Dongdong Zhang, Zhimei Lv

**Affiliations:** 1 Department of Nephrology, Shandong Provincial Hospital, Shandong University, Jinan, 250021 Shandong China; 2 Department of Nephrology, Shandong Provincial Hospital Affiliated to Shandong First Medical University, Jinan, 250021 Shandong China

**Keywords:** Diabetic nephropathy, Mechanisms of disease

## Abstract

Diabetic kidney disease, known as a glomerular disease, arises from a metabolic disorder impairing renal cell function. Mitochondria, crucial organelles, play a key role in substance metabolism via oxidative phosphorylation to generate ATP. Cells undergo metabolic reprogramming as a compensatory mechanism to fulfill energy needs for survival and growth, attracting scholarly attention in recent years. Studies indicate that mitochondrial metabolic reprogramming significantly influences the pathophysiological progression of DKD. Alterations in kidney metabolism lead to abnormal expression of signaling molecules and activation of pathways, inducing oxidative stress-related cellular damage, inflammatory responses, apoptosis, and autophagy irregularities, culminating in renal fibrosis and insufficiency. This review delves into the impact of mitochondrial metabolic reprogramming on DKD pathogenesis, emphasizing the regulation of metabolic regulators and downstream signaling pathways. Therapeutic interventions targeting renal metabolic reprogramming can potentially delay DKD progression. The findings underscore the importance of focusing on metabolic reprogramming to develop safer and more effective therapeutic approaches.

## Facts

- Metabolic reprogramming refers to the changes in cellular metabolic pathways that are necessary to support cell growth and proliferation.
- Metabolic reprogramming is characterized by mitochondrial biosynthesis dysfunction, increased glycolysis, and abnormal lipid and amino acid metabolism.
- Oxidative stress, inflammatory damage, abnormal autophagy and apoptosis caused by metabolic disorders contribute to the development of DKD and ultimately renal fibrosis.
- While current conventional treatments for DKD can slow down disease progression to some extent, they come with adverse effects, increased risk of cardiovascular disease, and eventual loss of renal function.

## Open questions

- Renal cells exhibit distinct physiological characteristics and functions. Is the mechanism of metabolic reprogramming consistent across these diverse cell types?
- DKD is a chronic pathological process, does the metabolic disorders of renal cells change as the disease progresses?
- Could it be a promising and safe therapeutic approach for managing DKD by regulating metabolic reprogramming and restoring normal mitochondrial function?

## Introduction

It is estimated that approximately 40% of diabetics can develop diabetic kidney disease (DKD), which is responsible for end-stage renal disease (ESRD) worldwide [1]. Metabolic disorders, such as hyperglycemia and hyperlipidemia, can damage renal cells through oxidative stress-mediated injury, inflammatory response, apoptosis, and autophagy, leading to renal tubulointerstitial inflammation and fibrosis, glomerular hypertrophy, glomerulosclerosis and ultimately renal insufficiency [2]. Metabolic reprogramming is a mechanism by which cells change metabolic patterns to meet energy needs for cell survival and growth [3]. Recently, it has been found that metabolic reprogramming was triggered by impaired mitochondrial biogenesis in a high-glucose environment and played a crucial role in the pathogenesis of DKD [4–6]. It is characterized by abnormal metabolism of the three major nutrients and suppression of mitochondrial oxidative phosphorylation (OXPHOS), which contributes to the onset and development of DKD. Kidney, a high energy-consuming organ, in which OXPHOS is inhibited in a hypoxic environment due to hyperglycemia. Consequently, the metabolic flux of glycolysis is increased to rapidly produce ATP to adapt to the changes [6, 7]. However, this compensatory response also causes damage to kidney. Additionally, abnormalities in fatty acid β-oxidation (FAO) and amino acid metabolism can lead to the buildup of lipids and uremic toxins in the kidney, which can contribute to renal cell damage and apoptosis [4, 8].

Unfortunately, the treatment of DKD still faces serious challenges. While conventional treatments, such as intensive glucose lowering and strict blood pressure control, can slow the decline in kidney function, they do not prevent the progression of renal failure. More studies have found two kinds of newer hypoglycemic agents, including SGLT2 inhibitors and GLP-1 agonists, can delay the progression of DKD by regulating metabolic reprogramming [9, 10]. However there are no more acknowledge about how great and safe impacts these drugs have on altering the progression of DKD. Therefore, there is an urgent need for new mechanisms leading to DKD and emerging treatment strategies.

This review discusses impacts of mitochondrial metabolic reprogramming in the context of diabetes on different types of renal cells, including renal tubular epithelial cells, podocytes, mesangial cells, and glomerular endothelial cells. We find that the end of metabolic reprogramming is cellular damage caused by oxidative stress, inflammation, resulting in development of DKD. The particular mechanism of metabolic reprogramming in the occurrence and progression of DKD needs to be further explored. This paper also summarizes various treatments of DKD, including traditional therapeutic agents and emerging therapeutic agents. Although the benefit of antihypertensive and hypoglycemic treatment is limited, the emerging medicine can delay or prevent ESRD through intervening in the process of metabolic reprogramming. Thus, in the future, modulating mitochondrial reprogramming and restoring substance metabolism will be promising treatment strategies.

## Mitochondrial metabolism

**Fig. 1:**
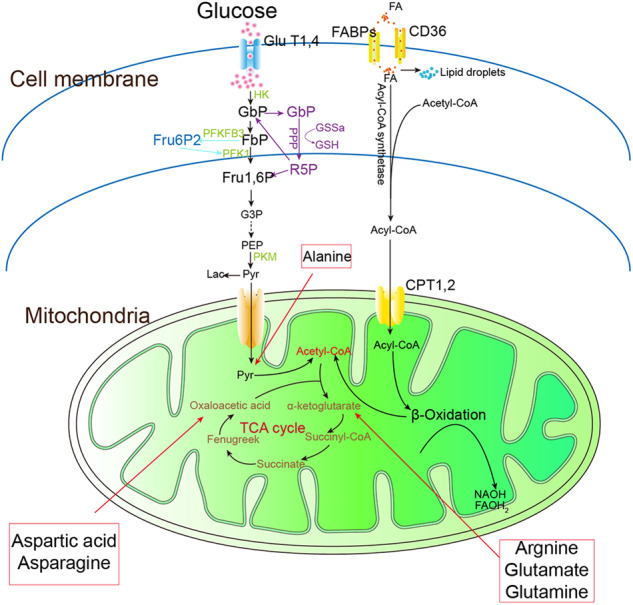
Glucose metabolism, fatty acid metabolism and amino acid metabolism in cells. GLUT1,4 glucose transporter1 and glucose transporter 4, G6P glucose-6-phosphate, F6P fructose-6-phosphate, Fru1,6P fructose 1,6-bisphosphate, HK hexokinase, PFKFB3 phosphofructokinase-2/fructose-2,6-bisphosphatase 3, PFK1 fructose-phosphate kinase-1, R5P ribose-5-phosphate, PPP pentose phosphorylation, G3P glyceraldehyde 3-phosphate, PEP phosphoenolpyruvate, Pyr pyruvate, TCA tricarboxylic acid cycle, NADPH nicotinamide adenine dinucleotide phosphate, FADH2 flavin adenine dinucleotide, PKM pyruvate kinase, Lac lactate, FA fatty acid, FABPs fatty acid-binding proteins, CPT1,2 carnitine palmitoyl transferase-1 and carnitine palmitoyl transferase-2.

Mitochondria, known as the energy converters and factories of the cell, play a critical role in aerobic oxidation and energy production. The metabolic processes of sugars, fatty acids, and proteins within mitochondria provide the cell with 95% of the energy needed for life activities (Fig. 1).

### Glucose metabolism

Glucose metabolism in the human body involves various pathways, including anaerobic and aerobic pathways, pentose phosphate pathway, glycogen synthesis, and catabolism pathway, and the glucuronide pathway. The aerobic oxidation pathway is the primary source of energy for cellular activities. Glucose is first converted to pyruvate in the cytoplasm, which is the first reaction of the aerobic oxidation pathway and also called glycolysis. Where the pyruvate goes depends on oxygen availability and mitochondrial respiratory capacity [11]. In normoxia, pyruvate enters cellular mitochondria through the mitochondrial pyruvate carrier (MPC) to begin the second reaction phase of the aerobic oxidation pathway. Pyruvate is oxidized and decarboxylated by the pyruvate dehydrogenase complex (PDC) in mitochondrial matrix to produce acetyl-CoA, as the beginning of the following tricarboxylic acid cycle (TCA cycle).

TCA cycle is a circular reaction system composed of a series of enzymatic reactions. The initial step in this process involves the catalysis of citrate synthesis from the condensation of acetyl-CoA with oxaloacetate by citrate synthase, which is considered one of the rate-limiting reactions of the TCA cycle. Subsequently, citrate is converted to isocitrate, which is then catalyzed by the rate-limiting enzyme isocitrate dehydrogenase to produce α-ketoglutarate. This compound can then be rapidly transformed by the α-ketoglutarate dehydrogenase complex into succinyl coenzyme A, with the concomitant production of NADH. Succinyl coenzyme A is further converted to succinate, which can generate GTP, and succinate is oxidized to fumarate and FADH2. Fumarate is then converted to malate, which is subsequently oxidized to oxaloacetate to initiate the next cycle of the TCA process. NADH and FADH2, as hydrogen carriers and electron donors, are produced during glucose metabolism. These hydrogen atoms and electrons can be transferred through the respiratory chain located in the inner mitochondrial membrane. This transfer process facilitates the reduction of oxygen molecules to water molecules and the phosphorylation of ADP to ATP. Ultimately, this leads to an OXPHOS reaction, providing energy for the organism. The TCA cycle is tightly regulated, with intermediates of the cycle exerting negative regulation on metabolic fluxes. Succinyl coenzyme A not only inhibits the activity of the α-ketoglutarate dehydrogenase complex but also citrate synthase. NADH inhibits all regulatory enzymes of the cycle, and ATP serves as a metabolic inhibitor for pyruvate dehydrogenase and isocitrate dehydrogenase complexes. The regulatory enzymes of the TCA cycle are activated under conditions of high ATP demand; conversely, in cases of impaired mitochondrial respiration, the accumulation of large amounts of NADH slows down the cycle [12]. In hypoxia, mitochondrial respiration is inhibited, the activity of related enzymes is reduced, and a large amount of pyruvate is stowed in the cytosol and converted into lactate.

### Lipid metabolism

The body’s fats are classified into two main groups: triglycerides (TG) and cholesterol. Triglycerides play a key role in energy metabolism, while cholesterol is primarily involved in cell membranes and hormone synthesis [13]. Fatty acids are key components of triglycerides and their catabolism, known as β-oxidation, is the primary mode of fatty acid metabolism within mitochondria [14]. In the presence of adequate oxygen, fatty acids are converted by acyl coenzyme A synthetase into acyl coenzyme A, which then undergoes a complex transport process into the mitochondria. Long-chain acyl coenzyme A requires carnitine palmitoyltransferase I (CPT1) on the outer mitochondrial membrane (OMM) to generate lipoylcarnitine with carnitine. This complex then crosses the inner mitochondrial membrane via translocase (CACT), which releases carnitine and regenerates lipoyl coenzyme A with the help of CPT2. CPT1 is the rate-limiting enzyme in this process, determining the rate of FAO. Malonyl coenzyme A negatively regulates CPT1, slowing down FAO by inhibiting its activity. Insulin indirectly counteracts this inhibition by promoting the synthesis of acetyl coenzyme A carboxylase (ACC), increasing malonyl coenzyme A levels. Short- and medium-chain fatty acids can directly enter mitochondria. Once in the mitochondrial matrix, lipoyl coenzyme A undergoes a 4-step reaction catalyzed by a fatty acid β-oxidase system, involving dehydrogenation, hydration, dehydrogenation, and sulfurolysis, ultimately producing acetyl-CoA. This acetyl-CoA is then utilized in the TCA cycle to generate ATP for the body’s energy needs.

### Protein metabolism

Proteins are vital for life processes and constitute a significant portion of the body’s tissues. They can be broken down in two ways: through proteolytic hydrolysis or proteasomal ubiquitination, leading to the breakdown of proteins into amino acids. In mitochondria, these amino acids can then be further metabolized by transaminases or deaminases to form keto acids, which are further oxidized to produce CO_2_ and H_2_O, along with ATP. Glutaminase catalyzes the conversion of glutamine to glutamate and ammonia, followed by the conversion of glutamate to alpha-ketoglutarate by either glutamate dehydrogenase or transaminase. Additionally, alanine and aspartate are deaminated to generate pyruvate and oxaloacetate, which serve as intermediates of the TCA cycle. Amino acids play a key role in signaling and metabolic regulation in the body. The breakdown of amino acids not only sustains the TCA cycle but also provides energy when glucose utilization is low.

The kidney, characterized by a high resting metabolic rate second only to the heart, exhibits significant mitochondrial content and oxygen consumption to support the energy-intensive process of urine production [15]. Various regions of the kidney carry out distinct physiological functions with varying energy requirements, contributing to a diverse metabolic profile within the organ. Renal ATP production primarily relies on oxidative phosphorylation of major nutrients in the mitochondria, supplemented by a minor contribution from glycolysis. The proximal and distal renal tubules in the renal cortex are abundant in mitochondria, serving as the primary site for fatty acid oxidation. Podocytes contain a moderate density of mitochondria that engage in both oxidative phosphorylation and glycolysis to meet energy demands, while the collecting ducts in the renal medulla have fewer mitochondria and typically rely more on glycolysis [16, 17]. To sustain continuous urine production, every cell in the kidneys undergoes metabolic reprogramming to meet energy demands, particularly in the early stages of DKD.

## Metabolic reprogramming of DKD

Kelli et al. utilized a systematic approach of transcriptomics analysis to evaluate glucose metabolism in diabetic mouse kidney cortical tissue. The results showed a significant increase in mRNA expression of key glycolytic enzymes such as HK1, PFK and PK, while the mRNA expression of enzymes involved in the TCA cycle pathway did not change significantly. They also found an upregulation of the expression of intermediates of glycolytic metabolism (hexose 6-phosphate and pyruvate) and intermediates of the TCA cycle (fumarate and malate) under metabolomics and metabolic flux analyses [18]. The high-glucose environment contributes to increased glucose uptake by the kidney and a significant increase in glycolytic flux. DKD experiences in a hypoxic environment that leads to the anaerobic glycolytic pathway for rapid ATP production to meet the high metabolic activity of the kidney [19].

Dyslipidemia is very common in patients with diabetes [20]. Research has shown that dyslipidemia, characterized by high levels of cholesterol and triglycerides, as well as impaired lipoprotein metabolism, including elevated very low density lipoprotein cholesterol and low density lipoprotein cholesterol, and reduced high density lipoprotein cholesterol, is associated with the development and progression of DKD [20]. A phenomenon that excessive deposition of elevated serum cholesterol and triglycerides in non-adipose tissues known as lipotoxicity [21]. And this condition may be caused by a mismatch between increased lipid uptake, decreased catabolism and efflux. Herman et al. showed a demonstration of significant lipid accumulation in renal tissues of diabetic patients. They determined regulation of fatty acid metabolism enzymes and cholesterol metabolism enzymes in diabetic kidney. Overexpression of CD36, SR-A1, LOX-1 and LDLR, which are transporters or receptors to mediate fatty acid and cholesterol uptake, reduction of ACO1, CPT1, which are involved in FAO, and low level of expression of ABCA1, which mediates cholesterol efflux, proved catabolism of lipids was inhibited in human diabetic kidney [22]. However, Kelli et al. utilized a systematic approach of transcriptomics, metabolomics, and metabolic flux analyses in mouse diabetic kidney cortex to demonstrate an increase in acylcarnitines, which can transport fatty acids to mitochondria as energy source. And they indicated the increase in metabolism in DKD was the result of an increased need for energy or adaptation to consumption of excess metabolic substrates [18]. The completely opposite results of these two studies may be due to differences in early and advanced DKD. Pathological development of DKD is a long process. Metabolic reprogramming, as one of the important pathogenetic mechanisms of DKD, needs to be further investigated.

The kidney acts as a clearance organ in the body and helps regulate synthesis and catabolism of amino acids through glomerular filtration and renal tubular reabsorption and secretion. Renal regulation of amino acids relies on amino acid transporter proteins. Disorders in amino acid metabolism and mutations in these transporter proteins can result in the development of aminoaciduria [23]. And amino acids are important signaling molecules that regulate energy and metabolic homeostasis as a basic components of proteins. The link between amino acid metabolism and insulin resistance has been recognized for decades [24]. In a cross-sectional study, Zhu et al. confirmed impaired amino acid metabolism was an important metabolic feature in the development of diabetes. They found an increase of N-acetylaspartic acid, L-valine, asparagine, betaine, and L-methionine could be metabolic markers of diabetes [8]. Tryptophan, an essential amino acid, plays a crucial role in the formation of various biologically active compounds. The KYN pathway serves as the primary metabolic route for tryptophan, facilitating its involvement in the TCA cycle and the production of NAD+ [25]. Studies have revealed that the expression of P5P phosphatase, a critical enzyme in the KYN pathway, is suppressed in DKD, resulting in the accumulation of KYN and its metabolites in the kidney [26]. Hyperglycemia causes Imbalance of gut microorganisms, intestinal barrier dysfunction and increased permeability of the intestinal mucosa, resulting in leaky gut [27]. IMP and IS, produced by the breakdown of histidine and tryptophan by intestinal microflora, are among the important sources of uremic toxins [28]. Therefore when kidney is dysfunctional, it can impact the synthesis and catabolism of amino acids. In turn, this disturbance in amino acid metabolism can further strain the kidney, leading to renal injury and creating a vicious cycle.

Mitochondria are semi-autonomous organelles with an autotrophic system. They undergo a dynamic process of fusion and fragmentation, known as mitochondrial dynamics, to maintain internal structure stability, eliminate damaged mitochondria, and meet cellular metabolic demands. This process involves transitioning mitochondrial morphology between an elongated network and a small spherical structure. Changes in bioenergetic demands can affect mitochondrial morphology by modulating the activity of mitochondrial dynamics proteins through metabolic pathway-related enzymes and signaling pathways, such as deacylase Sirtuin (SIRT) and mTOR signaling pathways [29, 30]. These pathways influence the activity of fusion proteins (dynamin-related protein 1) and segregation proteins (mitofusin 1/2 and optic atrophy type 1), as well as mitochondrial morphology [31]. Fusion between mitochondria enables mixed complementation of mitochondrial DNA (mtDNA) and inner mitochondrial membrane (IMM) respiratory proteins, ensuring mtDNA and ETC integrity, enhancing cellular OXPHOS activity, and meeting high energy demands [32]. On the other hand, mitochondrial division can trigger mitochondrial redox signaling by producing ROS to promote glycolytic conversion [33]. Studies have shown that exposure to high glucose or palmitate can lead to the downregulation of MFN1 and upregulation of DRP1 in renal cells, indicating altered mitochondrial dynamics and increased mitochondrial division [34]. Patients with DKD also exhibit increased mitochondrial division, resulting in mitochondrial fragmentation, uneven mtDNA distribution, reduced expression of enzyme complexes in the ETC, decreased OXPHOS activity, and enhanced glycolysis as a compensatory mechanism [35, 36]. However, the specific mechanisms through which altered mitochondrial dynamics regulate DKD metabolism remain unknown and require further investigation.

### Metabolic alterations in tubular epithelial cell

The kidney is one of the largest oxygen-consuming organs in the organism after the heart, in which the renal tubular epithelial cell (TEC) is the core site of energy metabolism in the kidney and requires large amounts of energy to sustain their reabsorption function [4]. In normal conditions, TECs primarily depend on FAO to produce substantial amounts of ATP to fulfill their physiological requirements. Non-adipocyte fatty acids are mediated primarily through the transmembrane protein CD36, fatty acid transport proteins (FATPs), and fatty acid-binding proteins (FABPs). Subsequently, fatty acids are transferred into the mitochondria for FAO catalyzed by CPT1 and CPT2. This process produces Acetyl-CoA, which then participates in the TCA cycle to supply energy to the kidney. In addition, acetyl-CoA generated by FAO in mitochondria can also be transported out via CACT and then re-synthesized into new fatty acids via ACC. Under DKD disease conditions, the energy metabolic pattern of TECs is altered and metabolic reprogramming occurs to provide energy and counteract oxidative stress. In brief, a high glucose environment induces a metabolic switch from fatty acid oxidation to glycolysis in renal proximal tubular epithelial cells (PTECs) [4, 18].

On the one hand, TECs can cause lipid accumulation and lipotoxicity due to the dysregulation of lipid metabolism. This dysregulation can occur through FAO inhibition, disturbed lipid uptake, or increased synthesis, among other factors. Ultimately, this reprogramming of lipid metabolism leads to renal TECs apoptosis, tubular atrophy, and tubulointerstitial damage. Notably, Studies have shown that HIF-1α is barely expressed in healthy kidney, but it is overexpressed in renal TECs in DKD [7]. HIF-1α, as a transcriptional regulator plays a key role in the dysregulation of peroxisome proliferator-activated receptor alpha (PPARα) and Acyl-CoA, which ultimately hinders the production of FAO [37, 38]. TGF-β1 is an important pro-fibrotic cytokine that regulates FAO [39]. Studies have shown that activation of the TGFβ1/Smad3 signaling pathway can decrease the expression of PGC-1α, which in turn reduces PPARα activity and promotes the deposition of fatty acids [40]. PPARα activator MHY2013 significantly reduced lipid accumulation in TECs and attenuated renal fibrosis in senescent rats [41], which further emphasized the importance of PPAR α and FAO in regulating renal lipid homeostasis. Moreover, ACC is a core enzyme involved in FAO and FA biosynthesis. Sterol regulatory element binding protein (SREBP) strictly regulates the expression of ACC to promote fatty acid synthesis [42]. Increased expression of SREBP-1 protein and concomitant lipid accumulation has been observed in both DKD patients and mouse models [43]. Liptin-1 deficiency in TECs can promote lipid synthesis and exacerbate renal tubulointerstitial fibrosis in DKD by inhibiting PGC-1α/PPARα and up-regulating SREBPs [44].

Lipid transporter protein disrupts TECs lipid uptake, exacerbating lipid metabolism disorders. Studies have demonstrated that HG can exacerbate lipid deposition in renal TECs by activating the AKT-PPARγ signaling pathway, which leads to an increase in CD36 expression [45]. In a high glucose environment, CD36 is upregulated, leading to a shift in the metabolic profile of TECs from FAO to glycolysis. This shift results in increased production of mitochondrial ROS, which in turn activates the NLRP3 inflammatory pathway, ultimately leading to inflammation and apoptosis in the kidney [46, 47]. The advanced oxidation protein products (AOPP), elevated in the plasma of patients with DKD, have been found to bind to the CD36 receptor, which can activate the Wnt/βcatenin signaling pathway as a result of lipid accumulation [48]. The accumulation of lipids in tubular cells leads to the differentiation of cells into a pro-fibrotic phenotype, which is a crucial factor in the development of renal fibrosis [39]. FATP2 is a crucial protein responsible for transporting fatty acids expressed in TECs. Studies on animal models of DKD have indicated that the uptake of non-esterified fatty acids through FATP2 leads to the accumulation of abnormal lipids, resulting in renal tubular atrophy [49]. The hyperactivation of FABPs may also contribute to the abnormal lipid uptake in DKD. Chen et al. discovered that renal biopsies of DKD patients showed increased FABP4 expression and decreased CPT1A expression. Inhibition of FABP4 expression in TECs led to elevated CPT1A expression, reversal of lipid deposition, and restoration of impaired FAO [50]. It is imperative to consider that intracellular lipid accumulation induces an increase in ROS production, mitochondrial dysfunction, and apoptosis, which further exacerbates the lipid excess. In turn, this leads to cellular differentiation into a pre-fibrotic phenotype, which is a crucial factor in the progression of renal fibrosis [39].

The disturbed lipid metabolism in TECs disrupts normal energy acquisition pathways, resulting in cytotoxic lipotoxicity injury. This also triggers compensatory glycolytic reactions, contributing to an acidic cellular environment. Therefore, regulating metabolic reprogramming and restoring normal lipid metabolism in TECs could significantly impact DKD therapy.

On the other hand, increased energy production from glycolysis leads to an increase in various metabolites and cellular secretion of various inflammatory and fibrogenic factors that promote renal tubular inflammation and fibrosis [51]. A recent study identified increased expression of the deacylase SIRT5 in proximal tubular epithelial cells. SIRT5 is responsible for removing malonylation from enzymes in the glycolytic pathway, thereby enhancing enzyme activity and promoting glycolysis. This metabolic switch in DKD may be explained by these findings [52]. Additionally, deacetylation of the mitochondrial protein NAD-dependent deacetylase SIRT3 plays a role in regulating mitochondrial metabolism. Unlike SIRT5, low expression of SIRT3 leads to increased acetylation and phosphorylation of PDHE1α, reduced PDHase activity, and enhanced glycolysis [53]. The excessive glycolysis and lactic acid accumulation in tubular epithelial cells result in lactylation of mitochondrial fission 1 protein (Fis1), leading to increased mitochondrial fission, mitochondrial ROS overproduction, and mitochondrial dysregulation [54]. Shao et al. observed that DKD patients exhibit significant downregulation of glycine metabolic pathways, reduced glutathione levels, a decreased glutathione to oxidized glutathione ratio, and elevated malondialdehyde, contributing to cellular oxidative stress. Supplementation with glycine has been shown to improve kidney injury by activating the AMPK/mTOR signaling pathway. Adenine has emerged as a potential predictor of DKD [55]. Purines, the building blocks of nucleic acids, are generated from the degradation of intracellular DNA, RNA, and ATP during cell death. Endogenous purines primarily originate from dead cells, and mitochondrial dysregulation leads to an accumulation of purines. Recent spatial metabolomics and single-cell transcriptomics studies by Kumar et al. revealed that endogenous adenine plays a role in the progression of renal disease in DKD patients by activating the mTOR pathway, which is crucial for cellular energetics pathways [56].

### Metabolic alterations in podocytes

Podocytes, along with glomerular capillary endothelial cells and the glomerular basement membrane (GBM), make up the glomerular filtration barrier. Podocytes are characterized by a stellate polypoid shape with numerous protrusions known as the foot process (FT). These foot processes cross the outer surface of the GBM in a finger-like pattern, and the fissures between them are called slit diaphragm (SD), which serve as the final line of defense for glomerular filtration. The contraction and expansion of the pedicles, which can be regulated by actin microfilaments, can alter the size of the lacunae and the area of the glomerular filtration membrane, thereby regulating the glomerular filtration rate. For podocytes, the high amounts of ATP are essential for regulating actin microfilaments and maintaining the glomerular filtration membrane [57, 58]. The main source of energy for podocytes is glucose metabolism, with both the anaerobic glycolytic pathway and mitochondrial OXPHOS providing energy for mature podocytes [59].

Notably, podocyte injury is an early event in the development of diabetic nephropathy. Diabetic kidney damage is usually associated with reduced numbers and loss of FT due to podocyte injury and is accompanied by disruption of the glomerular filtration membrane, leading to the development of massive proteinuria [60]. Imasawa et al. discovered that factors associated with mitochondrial biogenesis, such as transcription factor A (TFAM), PGC-1α, and nuclear respiratory factor 1 (NRF-1), were reduced under high-glycemic conditions, with a decrease in mitochondrial OXPHOS. This is when the podocyte undergoes a glycolytic metabolic switch and glycolysis is enhanced [61]. Pyruvate kinase M2(PKM2) has been focused as a key regulator of the process of podocyte development. It plays a significant role in controlling the differentiation of foot cells and can also reverse the harmful effects of hyperglycemia-induced mitochondrial dysfunction and toxic glucose metabolites [41, 49]. Additionally, Smad4 could be a potential therapeutic target for diabetic nephropathy. Li et al. discovered that high sugar concentrations caused an increase in Smad4 expression in the mitochondria of podocytes. This increase in Smad4 is directly bound to PKM2, resulting in reduced PKM2 activation and inhibition of podocyte glycolysis. The absence of smad4 was also found to enhance mitochondrial ATP synthase inhibitory factor (ATPIF1) activity, ATP synthase inhibition, and reduce mitochondrial membrane potential, ultimately promoting glycolysis by decreasing glucose metabolic flux and increasing glucose accumulation in the podocyte [62].

The glycolytic side-branch flow of podocytes is increased under high-glucose conditions. Additionally, activation of the polyol pathway can lead to oxidative stress damage and apoptosis in podocytes. Lanaspa et al. found significantly increased expression of aldose reductase, fructose, and uric acid in the kidneys of wild-type mice with diabetes induced by streptozotocin (STZ) [63]. Elevated glucose levels intensify oxidative stress through the upregulation of ROS production via multiple pathways including polyol, PKC, AGE/RAGE, and hexosamine pathways. This activation triggers pathways such as PI3K/Akt, TGF-β1/p38-MAPK, and NF-ΚB, resulting in endothelial cell apoptosis, inflammation, autophagy, and fibrosis. These processes contribute to histological and functional abnormalities in the kidney, culminating in kidney injury [64].

To compensate for the resulting lack of cellular energy, podocytes enhance their amino acid metabolism to increase intermediates of the TCA cycle and maintain OXPHOS. In a study, it was discovered that the upregulation of ornithine decarboxylase 1 (ODC1) and arginase 2 (ARG2), which are key enzymes in the urea cycle, led to enhanced ornithine catabolism and activation of the intracellular mTOR/ROCK1 signaling pathway in podocytes. This resulted in cytoskeletal remodeling and changes in cell morphology, ultimately leading to podocyte injury [65, 66].

Mitochondrial bioenergetics is closely associated with lipid metabolism, and lipid accumulation and metabolic dysregulation in podocytes lead to mitochondrial dysfunction, which results in podocyte injury. In diabetic nephropathy, up-regulated CD36 promotes free fatty acid (FFA) uptake, whereas down-regulated ABCA1 hinders extracellular transport of phospholipids and cholesterol. This blocks cholesterol efflux and leads to lipid deposition in the podocytes. As a result, mitochondrial dysfunction and generation of mitochondrial ROS, ultimately leads to podocyte damage [67]. Lipid rafts are regions of the cell membrane that are rich in cholesterol and are responsible for regulating the localization and function of proteins within the SD. These regions play a crucial role in regulating cellular signaling. However, excess intracellular cholesterol accumulation can interact with proteins in the SD, leading to changes in membrane potential and ion channels, which can ultimately affect podocyte function [68]. Palmitate is an FFA and another substrate for mitochondrial respiration. In addition, it has been shown that palmitic acid-induced lipotoxicity can cause podocyte damage. Wang et al. found that fatty acid oxidation regulates the sensitivity of podocytes to palmitic acid and is inversely related to acetyl-CoA carboxylase 2 (ACC2), a key enzyme of FAO [69]. In addition, the enhancement of FAO can be mediated by G protein-coupled bile acid receptor TGR5 agonists and accompanied by an increase in mitochondrial superoxide dismutase 2 (SOD2) activity, which reduces oxidative stress and lipid accumulation in the podocytes thereby attenuating the DKD injury [70].

### Metabolic alterations in glomerular mesangial cells

Glomerular mesangial cells (MCs) are intrinsic cells located within the glomerulus, positioned between the afferent and efferent arterioles. These contractile cells play a crucial role in regulating blood flow and glomerular filtration rate by adjusting capillary diameter. Additionally, MCs secrete various substances like ECM, TGF-β, and renin to maintain renal homeostasis. Recent research by Masanori et al. revealed that SGLT2 receptors on MCs act as glucose sensors, working in conjunction with Na^+^/Ca^2+^ exchangers to manage Na^+^ and Ca^2+^ levels, thereby influencing MCs contractility [71]. However, prolonged exposure to high glucose for 2 days resulted in loss of contractility and cell swelling in MCs, while cells treated with SGLT2 inhibitors maintained contractile function. Some scholars suggest that prolonged exposure to high glucose levels can lead to cell swelling through two main mechanisms. Firstly, extended high glucose exposure increases the activity of SGLT2, allowing a large influx of Na^+^ into the cell, ultimately causing cell swelling. Secondly, abnormal intracellular glucose metabolism activates the polyol pathway, leading to the accumulation of sorbitol and fructose, resulting in osmotic cell swelling [71]. In a high-glucose environment, SGLT2-mediated contractile dysfunction of MCs may contribute to hyperperfusion and hyperfiltration in DKD. Ang II is known to induce contraction in MCs. Research demonstrated that Ang II binding to its receptors on MCs up-regulates TRPC6, facilitating Ca^2+^ influx. This influx not only triggers cell contraction but also stimulates cell proliferation, up-regulates chemokines and fibroblast growth factors [72]. Activation of the polyol pathway depletes NADPH, leading to AGEs binding to their receptors on cell surfaces, resulting in oxidative stress, ROS generation, and activation of PKC and ERK1/2 signaling pathways, ultimately promoting TGF-β secretion, mesangial cell proliferation, and ECM synthesis, culminating in renal fibrosis [73]. ROS induced by high glucose levels activate the NF-ΚB pathway, leading to increased expression of EGR-1 and PKC-α in MCs of patients with DKD. This activation promotes localized inflammatory and fibrotic responses in the kidney by enhancing inflammatory factors MCP-1 and fibrotic markers such as collagen I and III [74]. Treatment with ACEIs or ARBs reduces EGR1 expression in glomeruli, inhibits renin expression, decreases secretion of TNF-a and fibronectin proteins (FN) from tethered cells, slows tethered cell injury, and affects the behavior of MCs [75].

MCs play a crucial role in phagocytosis and the removal of foreign bodies. In individuals with diabetes mellitus and hyperlipidemia, an abundance of foam cells has been identified in the tethered zone of their kidneys. These foam cells are generated through the phagocytosis of significant lipid quantities by MCs [76]. Research has indicated the presence of lipoprotein lipase (LPL) in MCs, serving as the primary enzyme for metabolizing lipoproteins. It was demonstrated that LPL amplifies triglyceride accumulation induced by very low density lipoprotein (VLDL) in MCs, leading to the ectopic deposition of large lipid amounts in the mesangium [77]. Consequently, the transition of MCs into foam cells results in the loss of contractile function, disrupting the balance of the glomerular microvascular environment and causing glomerular injury. Moreover, fatty acid-binding protein 4 (FABP4), a carrier protein for fatty acids, participates in fatty acid transport, metabolism, and signaling. Research has shown that dysregulated lipid metabolism and elevated plasma non-esterified fatty acids (NEFA) in DKD, such as palmitic acid and oleic acid, stimulate the upregulation of FABP4 in MCs, leading to apoptosis through endoplasmic reticulum stress [76]. Rho-associated, coiled-coil-containing protein kinase (ROCK), a serine/threonine kinase involved in fatty acid metabolism, has been observed to be significantly activated in DKD. Nagai et al. discovered that in mouse MCs, knocking down ROCK restored impaired fatty acid metabolism by modulating metabolic regulators like AMPK, PGC-1α, and CPT-1 [78].

Hyperglycemia can cause an imbalance in the intestinal bacteria and increase the permeability of the intestinal mucosal barrier. This can lead to the transfer of metabolites of intestinal microorganisms into the bloodstream, which can damage other organs [79]. For instance, the breakdown of histidine in the gut produces imidazole propionate (IMP), which accumulates in the kidney. IMP activates toll-like receptor 4 (TLR4) in MCs, leading to the activation of the inflammatory NF-κB signaling pathway and the release of inflammatory factors such as TNF-α, IL-6, and IL-1β. IMP also induces the expression of TGF-β1 and activates phosphorylation of Smad-3 and Erk1/2, contributing to inflammatory damage in MCs and renal fibrosis [80].

### Metabolic alterations in glomerular endothelial cells

The glomerulus is a capillary ball enclosed in a small kidney capsule and is an integral component of the kidney’s filtration system. The glomerular endothelial cells, which have multiple window pores, serve as the first line of defense of the glomerular filtration barrier. These cells are situated in the lumen of the ducts and have direct access to the blood. The endothelial glycocalyx (EG) is a complex structure made up of proteoglycans and glycosaminoglycans located in the apical membrane of endothelial cells. The glycocalyx serves as a natural barrier, safeguarding endothelial cells from blood cell adhesion stimuli. When disrupted, it can lead to direct damage to endothelial cells. Research indicates that in a high-glucose environment, the synthesis of glycocalyx components is hindered due to the stimulation of inflammatory mediators and ROS secretion. This disruption alters the structure of glycocalyx and reduces its quantity in diabetic mice [81]. In DKD, the expression of VEGF is reduced due to damage to podocytes. VEGF secreted by podocytes is responsible for binding to VEGFR2 expressed by glomerular endothelial cells, which helps maintain the structural and functional integrity of endothelial cells. Additionally, AGEs activate the TGF-β signaling pathway and inhibit endothelial cell VEGFR2 expression, leading to endothelial cell injury [82]. NO, a vasoactive substance released from vascular endothelial cells, plays a crucial role in regulating the growth and maintaining the integrity of the vascular endothelium. However, in a high glucose environment, AGEs and ROS-induced oxidative stress can inhibit the formation of nitric oxide synthase (NOS) dimers in endothelial cells, leading to reduced phosphorylation, NO release, and bioavailability. This, in turn, can cause vascular endothelial cell injury and apoptosis [83]. Song et al. discovered that elevated glucose levels increased the expression of insulin-like growth factor binding protein 5 (IGFBP5) in endothelial cells, resulting in the upregulation of EGR1. The key glycolytic enzyme phosphofructose-2-kinase/fructose-2,6-bisphosphatase 3 (PFKB3) primarily facilitates the synthesis of F-2,6-BP, thereby enhancing the activity of glycolytic enzyme PFK-1 and glycolysis. Moreover, EGR1 was found to boost the enzymatic function of PFKB3, leading to the secretion of inflammatory factors like ICAM-1, TNF-α, IL-6, and MCP-1 from endothelial cells through enhanced glycolysis, ultimately causing inflammatory damage [6].

Due to renal hyperperfusion, hyperfiltration, and hypoxia in a high-glucose environment, mitochondrial metabolic reprogramming occurs to meet cellular energy demands temporarily. However, this overcompensation leads to cellular lipid deposition, accumulation of metabolites, and activation of metabolic bypass pathways. These factors induce mitochondrial oxidative stress, activate signaling pathways like TGFβ/Smad and PKC/ERK, and stimulate the secretion of damaging cytokines and inflammatory mediators. This results in cellular inflammatory injury, apoptosis, and fibrosis. Subsequently, these cellular injuries impact metabolic transcription factors and regulate cellular metabolism, creating a harmful cycle.

## Therapeutics for regulating metabolic reprogramming in DKD

A 21-year follow-up study has explored the effects of intensive multifactorial therapy on the renal prognosis of diabetic patients with microalbuminuria. They found that early intervention in the form of strict control of blood glucose, lipids, and blood pressure, along with correction of dietary habits to control fat intake and long-term physical exercise can improve several indicators of kidney prognosis, including macroalbuminuria, GFR decline rate, ESRD and death [84]. However, it is important to note that this study was limited to patients with early diabetic nephropathy. The results of the intensive multifactorial treatment were found to be less effective for diabetic patients with high levels of proteinuria or challenging to manage blood pressure and blood glucose [85]. Recent research has focused on understanding the mechanisms through which metabolic reprogramming in DKD leads to kidney damage, and has shown that DKD can potentially be improved by modifying this metabolic reprogramming [86] (Table 1).

**Table 1:** Summary of therapies modulating different pathway of metabolic reprogramming against DKD.

Therapy	Metabolism	Target	References
Regular exercise	Glucose metabolism	AMPK and mTORC1	[91]
ACEI	OXPHOS	SIRT3, CPT1 and PGC-1α	[96, 97]
	FAO	Acetyl CoA carboxylase and PPARγ	[98, 99]
SGLT2 inhibitors	FAO	SIRT3 and HIF-1α	[102]
	Amino acids metabolism	mTORC1	[103, 104]
GLP-1 agonists	Lipid metabolism	ABCA1	[106, 107]
Traditional Chinese medicine	Lipid metabolism	SREBP-1, ACC and FAS	
	Lipid metabolism	PGC-1α, ABCA1 and APOE	[113]
	FAO	PGC-1α	[114]
Emerging drugs	Amino acids metabolism	Branched-chain amino acids and citrulline	[115]
	OXPHOS	PGC-1α and MPC	[116]
	FAO	PPARα	
	OXPHOS	VEGF-B	
	Glucose metabolism	Glucokinase activator	

### Non-pharmacological treatment

Adhering to dietary restrictions and engaging in regular exercise can effectively improve various metabolic disorders. Of the various lifestyle interventions, some intensity of physical activity is the simplest and most popularly accepted treatment. Regular exercise can also stimulate the hydrolysis and oxidation of fat, reducing lipotoxicity and promoting glucose homeostasis while reversing insulin resistance [87–89]. Kuo et al. demonstrated in a cross-sectional study that diabetics who engaged in ≥150 min of exercise per week had lower proteinuria [90]. Recent studies have also suggested that the protective effect of regular exercise on the kidneys is not dependent on blood glucose levels. Monno et al. showed that regular exercise in diabetic mice improved kidney injuries without affecting glucose concentration or insulin status. They proposed that regular exercise may restore autophagy and mitochondrial homeostasis in renal cells, while also reducing excessive apoptosis in the renal cortex through AMPK activation and mTORC1 inhibition [91].

Nutritional treatments for DKD remain a topic of ongoing debate among scholars. The prevailing belief is that a combination of nutritional therapy, regular exercise, and medication constitutes the most standardized treatment plan [92]. An inappropriate diet can exacerbate renal excretion and elevate the risk of cardiovascular disease in individuals with diabetes [93]. Research indicates that a low-protein diet, when combined with essential amino acids and ketoacids, may decelerate the progression of DKD, provided that intestinal flora balance is maintained, the intestinal barrier is protected, and the production of harmful metabolites and leaky gut are minimized [94]. However, for patients undergoing dialysis, significant protein loss makes adherence to a low-protein diet challenging [95]. Current nutritional therapy programs lack clear guidance, and due to individual patient variations, specific nutritional therapy plans cannot be universally recommended.

### Renin-angiotensin-aldosterone system (RAAS) inhibitors

Blood pressure control, particularly through the use of RAAS inhibitors like ACEI and ARBs, is a common treatment for DKD. These first-line drugs have been shown to effectively reduce the incidence of ESRD. Additionally, ACEI drugs have been found to be more nephroprotective than ARBs [96]. Nagai et al. demonstrated that the endogenous tetrapeptide N-acetyl-serinyl-aspartyl-lysinyl-proline (AcSDKP) has the ability to restore protein levels of SIRT3, CPT1a, and PGC1α and inhibit the expression of GLUT1, PKM2, and PDK4. AcSDKP was observed to restore mitochondrial OXPHOS and normalize substance metabolism in renal cells [97]. Furthermore, AcSDKP functions as an alternative substrate specifically for ACE enzymes, as it can only be hydrolyzed by them. The intake of ACEI drugs leads to an elevation in the level of AcSDKP within the body [96]. In view of this, the involvement of ACEI analogs in the regulation of renal metabolic reprogramming is achieved through the protective effect of AcSDKP on mitochondria.

Some recent studies discovered that ACEI analogs play a role in regulating lipid metabolism. These studies observed a notable decrease in lipid levels and reduced expression of ACC and PPARγ in mice treated with ACEI drugs, both of which are key players in fatty acid synthesis [98, 99]. However, conflicting studies have suggested that ACEI drugs may not have a significant impact on blood lipids [100]. Currently, there is limited knowledge about the role of ACEI analogs in regulating substance metabolism, indicating the need for further research.

### Glucose transporter protein 2 (SGLT2) inhibitor

SGLT2, which is primarily found in the proximal tubular epithelium, is responsible for glucose reabsorption. SGLT2 inhibitors work by reducing glucose reabsorption in the renal tubules, leading to lower blood glucose levels. Along with their ability to lower blood glucose, SGLT2 inhibitors have also been discovered to have nephroprotective properties that are independent of their glucose-lowering effects. These inhibitors activate adenosine A2 receptors, which dilate glomerular efferent arteries, reduce intra-glomerular hypertension, lower GFR, and decrease proteinuria and renal oxygen consumption [101]. In their study, Li et al. discovered that empagliflozin has the ability to restore SIRT3 expression in PTECs. This, in turn, can lead to a reduction in HIF-α levels, inhibition of the transition from FAO to abnormal glycolysis, and prevention of TECs from developing EMT. As a result, empagliflozin may help improve renal fibrosis [102].

Recent studies have shown that SGLT2 inhibitor drugs play a role in regulating amino acid metabolism in DKD. Kogot et al. discovered in mice that dapagliflozin can reduce renal fibrosis by suppressing the abnormal expression of collagen and amino acid transport proteins via mTORC1 inhibition [103]. Lu et al. conducted a 12-week study administering empagliflozin to db/db mice, resulting in decreased levels of KYN and increased expression of acetyl-CoA and NAD+ in the kidneys. The researchers proposed that empagliflozin may activate the crucial enzyme for NAD+ synthesis, potentially restoring the tryptophan metabolic pathway in DKD [104].

### Glucagon-like peptide-1 receptor (GLP-1R) agonist

Glucagon-like peptide-1(GLP-1), an enteroglucagon hormone produced by intestinal L-cells, activates the Glucagon-like peptide-1 receptor (GLP-1R) to enhance insulin secretion and inhibit glucagon secretion in a glucose concentration-dependent manner. Additionally, it can delay gastric emptying and reduce food intake through central appetite suppression, ultimately leading to lower blood glucose levels. Recent studies have also shown the presence of GLP-1R in kidney cells, where chronic high glucose stimulation may lead to GLP-1R ubiquitination and subsequent kidney cell damage [105].

Recent studies have shown that GLP-1 agonists play a role in regulating lipid metabolism. Some meta-analysis by Yao and Sun et al. revealed that GLP-1 agonists have notable lipid-lowering effects compared to glucose-lowering drugs like insulin, including reducing LDL, cholesterol, and triglycerides [106]. It has been proposed that GLP-1 agonists may hinder lipase secretion in the intestinal lumen, thereby slowing gastric emptying and inhibiting fat absorption [107]. Exendin-4, as a GLP-1R agonist, inhibits ERK1/2 by activating the PI3K/AKT signaling pathway. In addition, it increases the phosphorylation of AMPK and ACC and promotes the expression of PPAR-α and CPT1, which not only promotes lipolysis and inhibits adipogenesis, but also exerts an anti-inflammatory effect in glomerular endothelial cells [108]. Further research is needed to fully understand the mechanisms of GLP-1 agonists in lipid absorption and metabolism.

### Traditional Chinese medicines

Traditional Chinese medicine (TCM) has a long history in treating DKD, either alone or in combination with conventional drugs like ACEI/ARB’s, showing positive therapeutic effects [109, 110]. Recent studies have demonstrated that biologically active phytochemicals in TCM can modulate metabolic reprogramming in DKD with minimal adverse effects in clinical settings [111]. For example, the Zhenqing recipe, containing Fructus Ligustri Lucidi, Eclipta Prostrata, and Dioscorea opposite, was found to inhibit the expression of SREBP-1C and its target genes, ACC and FAS, in diabetic rats, leading to a significant reduction in triglycerides and cholesterol levels [112]. Morroniside, an iridoid glycoside from Cornus officinalis Sieb, was shown by Gao et al. to up-regulate the expression of PGC-1α, LXR, ABCA1, and ApoE, promoting intracellular cholesterol efflux in the kidneys of diabetic mice [113]. Qin et al. discovered that berberine has the ability to activate the PGC-1α/LXR signaling pathway in db/db mouse foot cells. This activation restores mitochondrial homeostasis, normalizes fatty acid oxidation, reverses metabolic disorders, and repairs cellular damage [114]. The precise targeting mechanism of Chinese herbs for DKD requires further investigation. Current research indicates that various biologically active plant components found in Chinese herbs show promise in treating DKD effectively. However, a deeper understanding of the therapeutic mechanisms of these components is necessary to achieve precise treatment of DKD using Chinese herbs.

### Emerging drugs

With the advancing comprehension of mitochondrial metabolic reprogramming, there is a growing number of targeted therapeutic agents being uncovered. Recent studies have shown that artemether treatment in diabetic mice led to decreased levels of branched-chain amino acids and citrulline, indicating a potential alteration in amino acid metabolism in DKD [115]. Additionally, artemether was found to upregulate the expression of PGC-1α and MPC in the renal cortex of diabetic mice, enhancing pyruvate oxidation in the mitochondrial matrix and regulating mitochondrial function to ameliorate renal injury [116]. Clinical trials focusing on targeted drug therapies for metabolic reprogramming have been emerging. For instance, fenofibrate (NCT03869931), a PPARα agonist, has been shown to restore fatty acid oxidation and is commonly used for hypertriglyceridemia treatment. Annelie et al. discovered that inhibition of VEGF-B signaling reduced lipid accumulation in the kidney, mitigated renal lipotoxicity, and prevented renal dysfunction [117]. The effectiveness of 2H10 (NCT04419467), a monoclonal antibody targeting VEGF-B, is currently being investigated in patients with DKD. Furthermore, Dorzagliatin (NCT06222476), a glucokinase activator serving as a novel hypoglycemic agent, aids in enhancing the body’s intrinsic capacity to regulate glucose levels.

The treatment program for DKD is now more advanced, incorporating lifestyle improvements alongside symptomatic medications to regulate blood glucose, blood lipids, and blood pressure. In cases of end-stage DKD, renal replacement therapy is the only option. With the discovery of metabolic reprogramming in DKD and its impact on kidney damage, there is potential for drugs targeting metabolic reprogramming to halt disease progression at a pathogenic level.

## Conclusions

**Fig. 2:**
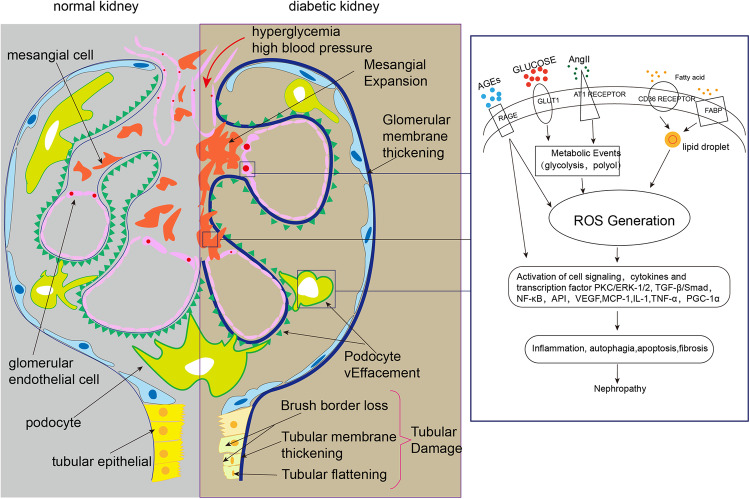
Overview of the pathogenesis of diabetic kidney disease (DKD). In a healthy kidney, the glomerular filtration barrier is formed by intact glomerular endothelial cells, basement membranes, and podocytes. This barrier effectively prevents the filtration of macromolecules like plasma albumin from the blood. The contractile action of mesangial cells regulate the tubular diameter of the afferent and efferent arterioles, thereby controlling the glomerular filtration rate. Additionally, mesangial cells support the position of vascular collaterals. Tubular epithelial cells play a role in regulating the formation of primary urine through functions like reabsorption, secretion, and excretion. However, hyperglycemia-induced changes in kidney hemodynamics, activation of the RASS, generation of AGEs leading to mitochondrial metabolic reprogramming, and excessive release of ROS trigger a cascade of signaling pathways, cytokines, and transcription factors. These factors contribute to the development of various pathological responses in renal cells, including inflammation, autophagy, apoptosis, and fibrosis.

DKD is a glomerular disease that results from diabetes mellitus, and is classified as one of its microvascular complications. The increased sugar concentration in the blood causes hyperperfusion and hyperfiltration of the glomeruli, as well as excessive tubular reabsorption, leading to an increase in renal oxygen consumption. This altered hemodynamics in the kidney, along with activation of the local RAS system, can lead to vascular lesions and a reduction in blood vessels. Stimulation by high glucose and hypoxia can cause mitochondrial dysfunction in renal cells. This can lead to abnormal expression of regulatory factors, such as HIF-α and SIRT3, which can affect a range of signaling pathways and stimulate the onset of abnormal glycolysis and amino acid metabolism. Additionally, this can inhibit the FAO response, which can enhance lipotoxicity. The production of large amounts of ROS and inflammatory factors can result in oxidative stress damage, inflammatory damage, autophagy, and apoptosis in renal cells (Table 2). Ultimately, this can lead to renal fibrosis and renal dysfunction (Fig. 2).

**Table 2:** Summary of cellular injuries in DKD.

Type of kidney cells	Metabolic characteristics	Mitochondrial metabolism	Injuries	References
Tubular epithelial cells	TECs contain a large number of mitochondria and use FAO as the main source of energy.	Glucose metabolism	Inflammation	[51]
			Oxidative stress	[54]
			Apoptosis	[51]
			Autophagy disorder	[51]
		Lipid metabolism	Inflammation	[46, 47]
			Oxidative stress	[39, 46, 47]
			Apoptosis	[39, 48, 50]
		Amino acid metabolism	Oxidative stress	[55]
Podocytes	Podocytes contain a moderate density of mitochondria, which undergo both oxidative phosphorylation and glycolysis to satisfy the energy demand.	Glucose metabolism	Oxidative stress	[22, 41]
			Apoptosis	[64]
		Lipid metabolism	Oxidative stress	[67]
			Apoptosis	[68]
			Autophagy disorder	[68, 70]
		Amino acid metabolism	Oxidative stress	[65, 66]
Mesangial cells	MCs and glomerular endothelial cells have fewer mitochondria and rely primarily on glycolysis to maintain cellular function.	Glucose metabolism	Inflammation	[74]
			Oxidative stress	[73]
		Lipid metabolism	Inflammation	[74, 75]
			Apoptosis	[76]
		Protein metabolism	Inflammation	[80]
Glomerular endothelial cells		Glucose metabolism	Inflammation	[6, 81]
			Oxidative stress	[81, 83]
			Apoptosis	[83]

DKD is a condition in which high blood glucose levels lead to a series of kidney damages. Lowering blood glucose levels alone cannot prevent the development of DKD. The pathogenesis of DKD is complex, as it involves extensive damage caused by the reprogramming of mitochondrial metabolism in kidney cells. Therefore, treatment for DKD should focus on correcting mitochondrial metabolic reprogramming and restoring normal glucose and fatty acid metabolism, in addition to controlling blood glucose levels. Understanding the role of mitochondrial metabolic reprogramming in various cells during the development of DKD is crucial for comprehending the disease’s pathogenesis and developing more effective nephroprotective therapies.
